# Prediction contribution of the cranial collateral circulation to the clinical and radiological outcome of ischemic stroke

**DOI:** 10.1007/s00415-020-09798-0

**Published:** 2020-03-23

**Authors:** Julian Conrad, Matthias Ertl, Meret H. Oltmanns, Peter zu Eulenburg

**Affiliations:** 1 Department of Neurology, LMU Munich, Marchioninistr.15, 81377 Munich, Germany; 2 German Center for Vertigo and Balance Disorders (DSGZ), LMU Munich, Munich, Germany; 3 Department of Psychology, University of Bern, Bern, Switzerland; 4 Department of Neuroradiology, Johannes Gutenberg-University, Mainz, Germany; 5 Institute for Neuroradiology, LMU Munich, Munich, Germany

**Keywords:** Stroke, Collaterals, CT-angiography, Ischemic, Score

## Abstract

**Background and Aim:**

The extent of penumbra tissue and outcome in stroke patients depend on the collateral cranial vasculature. To provide optimal individualized care for stroke patients in the emergency room setting we investigated the predictive capability of a stringent evaluation of the collateral vessels in ischemic stroke on clinical outcome and infarct size.

**Methods:**

We retrospectively studied uniform clinical and radiological data of 686 consecutive patients admitted to the emergency department with suspected acute ischemic stroke. Cranial collateral vasculature status was graded using the initial CT-angiography. Outcome was measured by mRS, NIHSS and final infarct size at hospital discharge. All data were used to build a linear regression model to predict the patients´ outcome.

**Results:**

Univariate and multivariate analyses showed significant effects of the whole brain collateral vessel score on all outcome variables. Atherosclerosis and piale collateral status were associated with the final infarct volume (FIV). Atherosclerosis and age were associated with the NIHSS at discharge. The presence of atherosclerosis, glucose level on admission and age were associated with the mRS at discharge. The multivariate models were able to predict 29% of the variance of the mRS at discharge, 24% of the variance in FIV and 17% of the variance of the NIHSS at discharge. The whole brain collateral status and the presence of atherosclerosis were the most relevant predictors for the clinical and radiological outcome.

**Conclusion:**

The whole brain collateral vasculature status is clearly associated with clinical and radiological outcome but in a multivariate model seems not sufficiently predictive for FIV, mRS and NIHSS outcome at discharge in non-preselected patients admitted to the emergency department with ischemic stroke.

**Electronic supplementary material:**

The online version of this article (10.1007/s00415-020-09798-0) contains supplementary material, which is available to authorized users.

## Introduction

In ischemic stroke artery occlusion leads to the hypoperfusion of the subsequent areas within the territory of the occluded vessel. If perfusion is less than 10 mL/100 g/min, nerve cells are irreversibly damaged within minutes [1]. If cerebral blood flow is partially reduced nerve cells cease to function but are still structurally intact and are considered potentially salvable tissue which makes them the target of thrombolytic therapy [2]. Various studies have shown that patients with good collateral status have larger ischemic penumbra, smaller infarct size and better outcome following thrombolysis and recanalization procedures [3, 4].

We aimed to predict clinical outcome on the grounds of the general collateral vessel status on the initial CT-angiography to develop an overall score to identify patients with a low collateral status that might profit from a more aggressive interventional therapy thereby facilitating a more personalized care of stroke patients in general. CT-angiography was chosen because it is fast, easily applicable, widely available and does not require additional time-consuming image processing and reflects general medical practice.

## Methods

### Patients

We retrospectively studied the clinical and radiological data of 686 patients that were admitted to the tertiary referral center with an acute stroke and obtained a full radiological work-up [native CT, supra-aortal CT angiography (CTA) and a dedicated follow-up examination (cranial MRI or CT)] to quantify the final lesion size and location of the ischemic stroke (excluded: *n* = 156; data quality, *n* = 69; alternative diagnosis, *n* = 87, final analysis *n* = 530).

### Predictor-variables

The entire cranial collateral vasculature status of the patients was evaluated by applying an in-house scoring sheet to the initial CT-angiography. All vessels were graded by one experienced clinician who was blinded for the other data and diagnosis at the time of evaluation (Table 1).

**Table 1:** Grading score for the affected vascular territories

VA, BAS, PCA, Pcom, ICA, ACA, Acom, MCA:
− 4: occluded
− 3: visible stenosis
− 2: hypoplastic
− 1: diameter variations
0: not assessable
1: normal
PICA, AICA, SCA, ophthalmic artery, medial meningeal artery:
1: assessable
0: not assessable
Piale arteries cortical:
0: 0–8 pial arteries visible
1: 9–11 pial arteries visible
2: > 11 piale arteries visible
Piale cerebellar arteries:
0: not visible / not assessable
1: < 3 piale arteries visible
2: > 2 piale arteries visible

*VA* vertebral artery, *BAS* basilar artery, *PCA* posterior cerebral artery, *Pcom* posterior communicating artery, *ICA* internal carotid artery, *ACA* anterior cerebral artery, *Acom* anterior communicating artery, *MCA* middle cerebral artery, *PICA* posterior inferior cerebellar artery, *AICA* anterior inferior cerebellar artery, *SCA* superior cerebellar artery

For the comprehensive arterial vasculature status score of the brain the left and right sides were added together to obtain one value per vessel assuming that atherosclerotic changes are evenly distributed in both hemispheres. The scores of the anterior cerebral artery (ACA-), middle (MCA-), posterior (PCA-) segments were also summed up but an additional weighting factor was introduced as atherosclerosis located more proximal in the course of the vessel would produce a more devastating effect on clinical outcome. The ACA-segments were calculated as ACA = 2 × A1 + A2 while the other two were calculated as MCA = 4 × M1 + 2 × M2 + M3 and PCA = 4 × P1 + 2 × P2 + P3. The segments of the internal carotid artery were summarized in one ICA factor.

Relevant co-factors such as age, sex, atherosclerosis, infections, nicotine abuse and statin consumption, symptom onset, time to door and time to treatment were included. Atherosclerosis was recorded if CT angiography showed intravascular calcifications or hypodense plaques attached to the arterial wall. The applied intervention (none, iv thrombolysis, arterial thrombolysis or bridging lysis in combination with thrombectomy) and the infarct region were examined as well.

### Outcome-variables

Final infarct size, National Institutes of Health Stroke Scale Score (NIHSS), and modified Ranking Scale (mRS) at the time of discharge were used as dedicated outcome variables (median 8 days). The infarct size was manually measured based on the high resolution CT or diffusion-weighted MR images at least 48 h after symptom onset.

### Modelling

The data analysis and statistical modeling were performed using SPSS (Version 23.0.0.0, Armonk, NY, IBM Corp.). To test the effect of risk factors, demographic variables, collateral status and treatment on functional outcome univariate models were computed for each of the outcome variables (Cramers’ *V*, *χ*^2^ tests for metric variables, continuous variables were tested using Pearson’s *r*). To predict the outcome of the three variables linear regression models were applied. In a second analysis step, we used a MANOVA approach to account for possible interactions of the dependent variables. Initially, the entire dataset was analyzed using all available variables as predictors. In a second step, only MCA territory strokes and different stroke volumes were considered.

### Standard protocol approvals and patient consents

The study was performed in accordance with the 1964 Declaration of Helsinki and was approved by the local ethics committee. Written consent for data usage was obtained from all patients.

## Data availability

The dataset is not publicly available but can be obtained from the corresponding authors on reasonable request.

## Results

### Predictor-variables

**Fig. 1:**
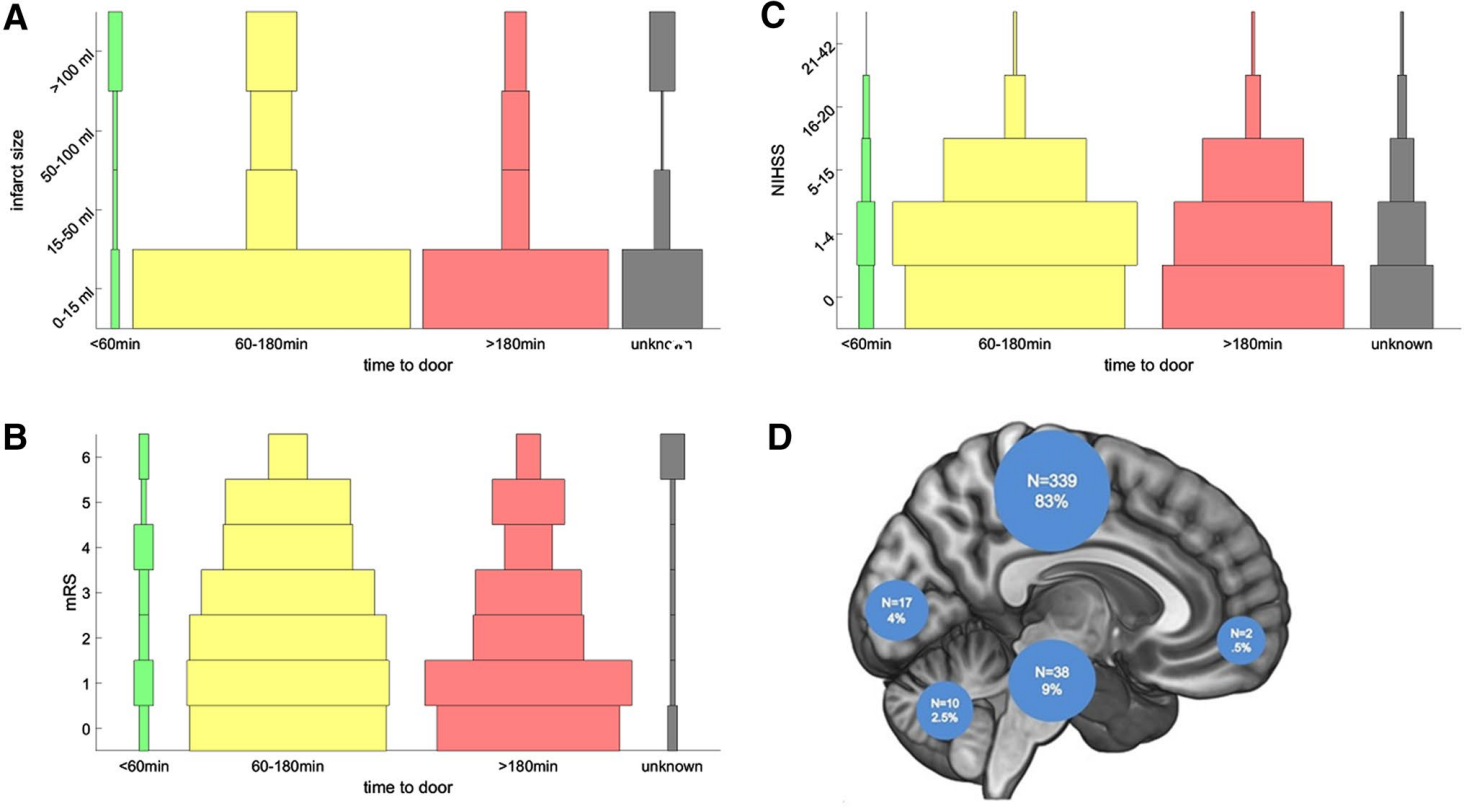
**a**–**c** show the effect of symptom duration before getting to the emergency department on FIV; NIHSS and mRS. **d** Relative distribution of the affected vascular territories

Demographic data are shown in Table 2, the distribution of vascular territories among the 530 patients is shown in Fig. 1d. In 91 patients the diagnosis was later changed clinically to transient ischemic attack (TIA).

**Table 2:** Demographics, cofactors and treatment outcomes

Age	65.7 years (± 15.0; range 19–90 years)		
Gender			
Female	207 (39.1%)		
Male	323 (60.9%)		
NIHSS admission (median, IQR)	6.0 (8.0)		
NIHSS discharge (median, IQR)	1 (4.0)		
mRS admission (median, IQR)	3.0 (2.0)		
mRS discharge (median, IQR)	2.0 (2.0)		
Infarct volume (median, IQR)	1.94 ml (27.8)		
Atherosclerosis (*n*/%)	175 (33%)		
Smoking (*n*/%)	207 (39.1%)		
Elevated glucose level on admission (*n*/%)	28 (5.3%)		
Infection (*n*/%)	13 (2.5%)		
Intervention (*n*/%)			
IV thrombolysis	171 (32.2%)		
IA thrombolysis (± bridging)	32 (6%)		
Mechanical thrombectomy	11 (2%)		
No intervention	292 (55.1%)		
Final infarct volume (FIV)			
< 15 ml	66.00%		
15–50 ml	11.70%		
50–100 ml	8.90%		
> 100 ml	13.30%		
Treatment outcomes (median, IQR)	FIV (ml)	NIHSS	mRS
IV thrombolysis	5.4 (56.68)	2.0 (6.0)	2.0 (3.0)
IA thrombolysis (± bridging)	77.1 (223.4)	3.0 (12.0)	4.5 (2.5)
Mechanical thrombectomy	36.6 (55.35)	5 (.5)	2.0 (4.0)
No intervention	0.56 (8.04)	1 (4.0)	1.0 (3.0)
MCA territory only			
LVOS	105 (31.0%, 90 M1 segment, 15 ICA)		
Final infarct volume (FIV)			
< 15 ml	61.70%		
15–50 ml	13.00%		
50–100 ml	9.00%		
> 100 ml	16.20%		
Treatment outcomes (median, IQR)	FIV (ml)	NIHSS	mRS
IV thrombolysis	4.86 (59.3)	2 (6.0)	2 (2.3)
IA thrombolysis (± bridging)	113.81 (222.4)	5.5 (13.3)	4.0 (3.0)
Mechanical thrombectomy	35.4 (44.5)	5 (6.0)	2 (3.3)
No intervention	1.2 (22.8)	1.5 (5.0)	2.0 (3.0)

### Outcome-variables

The median infarct volume (FIV) was 2.76 ml (median 1.94 ml), for the distribution of FIV across the patient population see Table 2. Of all patients, 3.2% were evaluated within the first hour of symptom onset. In 16.4% (87) of the patients the exact time of symptom onset could not be determined (Fig. 1a, b). A third of the patients (32.2%, *n* = 171) received iv thrombolysis with rt-PA, another 32% of the investigated cohort was treated with either intraarterial thrombolysis or in combination with thrombectomy (*n* = 3) or applying a bridging lysis regimen and thrombectomy (*n* = 9). More than half of the patients (55.1%, *n* = 292) did not receive any acute intervention.

The median NIHSS at onset was 6.0 and 1 at the time of discharge from the hospital. The initial mRS was 3.0 and 2.0 for the follow-up measurement at discharge.

The correlation between the mRS and the NIHSS was 0.634 (*p* < 0.001) and the correlation between mRS and infarct size was 0.622 (*p* < 0.001). The correlation coefficient of the NIHSS with infarct volume was 0.541 (*p* < 0.001).

### Univariate analysis

Univariate analysis showed an effect for the presence of atherosclerosis in the CTA on admission, elevated blood glucose level on admission, piale collateral status, baseline NIHSS and therapy on the FIV. Atherosclerosis, age, treatment, age and stroke severity were significantly related to NIHSS score at discharge. Finally, atherosclerosis, glucose level on admission, age and stroke severity were significantly associated with the mRS. The whole brain vessel score was correlated with all three outcome variables in univariate analysis when considering all patients as well as the subgroup of MCA territory strokes only. (Table 3). No significant effect on the three outcome variables was found for collateral status in severe stroke (i.e., NIHSS > 17, *n* = 36).

**Table 3:** Univariate analysis of co factors and collateral status on FIV, NIHSS and mRS at discharge (Cramers’ *V*, Pearson’s *r*, *p* < 0.05)

FIV	All patients		MCA territory only	
	Effect size	Sig	Effect size	Sig
	Cramers *V*	*p*	Cramers *V*	*p*
Atherosclerosis	0.122	**0.0094**	0.12	0.3
Infection	0.1	0.232	0.088	0.618
Glucose	0.18	**0.003**	0.177	**0.03**
Statine	0.066	0.638	0.107	0.471
Male sex	0.053	0.751	0.186	**0.019**
Smoking	0.032	0.937	0.157	0.096
	Pearson's *r*	*p*	Pearson's *r*	*p*
Age	0.39	0.39	0.011	0.853
Whole brain collateral vessel score	− 0.459	**< 0.0001**	− 0.38	**< 0.0001**
Piale collaterals only	− 0.209	**< 0.0001**	− 0.251	**< 0.0001**
Baseline NIHSS	0.59	**< 0.0001**	0.615	**< 0.0001**
NIHSS discharge	0.541	**< 0.0001**	0.423	**< 0.0001**
mRS discharge	0.622	**< 0.0001**	0.556	**< 0.0001**
	Cramers *V*	*p*	Cramers *V*	*p*
Therapy	0.414	**< 0.0001**	0.436	**< 0.0001**

NIHSS discharge	Cramers *V*	*p*	Cramers *V*	*p*
Atherosclerosis	0.151	**0.008**	0.156	**0.034**
Infection	0.059	0.474	0.093	0.298
Glucose	0.25	** < 0.0001**	0.221	**0.001**
Statine	0.04	0.722	0.076	0.449
Male sex	0.02	0.914	0.016	0.963
Smoking	0.039	0.957	0.054	0.672
	Pearson's *r*	*p*	Pearson's *r*	*p*
Age	0.125	**0.01**	0.108	0.074
Whole brain collateral vessel score	− 0.305	**< 0.0001**	− 0.33	**< 0.0001**
Piale collaterals only	0.037	0.46	− 0.1	0.109
Baseline NIHSS	0.677	**< 0.0001**	0.638	**< 0.0001**
mRS discharge	0.559	**< 0.0001**	0.539	**< 0.0001**
	Cramers *V*	*p*	Cramers *V*	*p*
Therapy	0.2	**0.029**	0.23	0.063

mRS discharge	Cramers *V*	*p*	Cramers *V*	*p*
Atherosclerosis	0.217	**< 0.0001**	0.25	**< 0.0001**
Infection	0.05	0.319	− 0.27	0.665
Glucose	0.122	**0.014**	0.122	0.052
Statine	0.054	0.284	− 0.059	0.349
Male sex	0.01	0.839	0.033	0.596
Smoking	0.099	0.139	0.005	0.924
	Pearson's *r*	*p*	Pearson's *r*	*p*
Age	0.208	**< 0.0001**	0.235	**< 0.0001**
Whole brain collateral vessel score	− 0.446	**< 0.0001**	-0.436	**< 0.0001**
Piale collaterals only	− 0.035	0.5	-0.133	0.04
Baseline NIHSS	0.636	**< 0.0001**	0.56	**< 0.0001**
NIHSS discharge	0.586	**< 0.0001**	0.539	**< 0.0001**
FIV	0.264	**< 0.0001**	0.387	**< 0.0001**
			Cramers *V*	*p*
Therapy	0.225	< 0.0001	0.177	0.09

### Regression models

We first estimated a linear regression model using whole brain collateral status score, piale collateral score, atherosclerosis, elevated blood glucose level on admission, statine medication, infectious constellation (clinical or laboratory tests), history of smoking, age, sex and the type of applied treatment as predictor variables. This first model including the whole dataset explained 29.0% of the variance when predicting the mRS, 24% when predicting the FIV and 17% when predicting the NIHSS. The predictive capacity changed only moderately when including only strokes in the MCA territory or medium volume strokes (15–100 ml). The whole brain collateral status was the strongest predictor for all outcome measures. Apart from the collateral status, the presence of atherosclerosis was predictive of the NIHSS at discharge in all models, while an elevated glucose level on admission and age were predictive of the mRS at discharge when considering the whole data set and medium to large (non-malignant) volume strokes (FIV 15–100 ml). We attempted to analyze the subgroup of malignant profile / severe strokes (i.e., infarct volume > 100 ml, NIHSS > 17), however, this analysis did not yield reliable enough results due to the small number of cases in the sub-analysis (see Table 4) [5].

**Table 4:** Multivariate linear regression models predicting the variance of FIV, NIHSS and mRS at discharge (p < 0.05)

All territories	Final infarct volume (FIV)				NIHSS discharge				mRS discharge			
	*t*	*p*	*ß*		*t*		*ß*		*t*	*p*	*ß*	
Whole brain vessel score	− 6.91	**< 0.001**	− 6.91		-5.62		− 0.30		− 7.19	**< 0.001**	− 0.37	
Piale collaterals only	− 1.94	0.053	− 1.94		-0.39		− 0.02		0.10	0.923	0.01	
Atherosclerosis	− 0.55	0.58	− 0.55		2.04		0.11		− 0.09	0.926	− 0.01	
Glucose level	0.34	0.738	0.34		2.47		0.12		2.13	**0.034**	0.10	
Statine	− 0.01	0.996	− 0.01		-0.65		− 0.03		− 1.36	0.174	− 0.06	
Smoking	1.76	0.079	1.76		0.51		0.02		1.37	0.173	0.07	
Infection	− 0.61	0.543	− 0.61		1.07		0.05		1.76	0.08	0.08	
Age	0.33	0.742	0.33		1.22		0.06		4.71	**< 0.001**	0.24	
Male sex	1.83	0.067	1.83		0.20		0.01		0.36	0.72	0.02	
IV thrombolysis	2.62	**0.009**	2.62		0.65		0.03		2.43	**0.016**	0.12	
IA thrombolysis (bridging)	4.77	**< 0.001**	4.77		0.90		0.05		1.96	0.051	0.10	
Thrombectomy	− 0.37	0.711	− 0.37		2.72		0.131		1.115	0.266	0.052	
Overall model	***F***	***df***	***p***	**adj R**^**2**^	***F***	***df***	***p***	**adj R**^**2**^	***F***	***df***	***p***	**adj R**^**2**^
	11.04	12,355	**< 0.0001**	**0.24**	7.224	12,361	**< 0.0001**	**0.17**	12.519	12,333	**< 0.0001**	**0.29**

MCA only	Final infarct volume (FIV)				NIHSS discharge				mRS discharge			
	*t*	*p*	*ß*		*t*		*ß*		*t*	*p*	*ß*	
WholeBrain vessel score	− 4.49	**< 0.001**	− 0.29		− 5.62		− 0.30		− 6	**< 0.001**	− 0.38	
Piale collaterals only	− 2.13	**0.03**	− 0.13		− 0.39		− 0.02		− 0.68	0.497	− 0.04	
Atherosclerosis	− 0.22	0.83	− 0.01		2.04		0.11		0.07	0.942	0.00	
Glucose level	1.15	0.25	0.07		2.47		0.12		2.11	**0.036**	0.12	
Statine	0.57	0.57	0.03		− 0.65		− 0.03		− 1.07	0.287	− 0.06	
Smoking	1.91	0.06	0.11		0.51		0.02		0.36	0.716	0.02	
Infection	− 1.11	0.27	− 0.07		1.07		0.05		0.85	0.396	0.05	
Age	0.21	0.83	0.01		1.22		0.06		4.72	**< 0.001**	0.31	
Male sex	2.98	**< 0.001**	0.18		0.20		0.01		0.78	0.437	0.05	
IV thrombolysis	1.14	0.26	0.07		0.65		0.03		− 0.38	0.705	− 0.02	
IA thrombolysis (bridging)	3.68	**< 0.001**	0.23		0.90		0.05		0.78	0.437	0.05	
Thrombectomy	− 0.61	0.541	− 0.04		2.715		0.131		− 0.04	0.968	− 0.002	
Overall model	***F***	***df***	***p***	**adj R**^**2**^	***F***	***df***	***p***	**adj R**^**2**^	***F***	***df***	***p***	**adj R**^**2**^
	6.25	12,228	**< 0.001**	**0.21**	7.224	12,361	**< 0.001**	0.17	8.571	12,215	**< 0.001**	**0.29**

Malignant (FIV > 100 ml)	NIHSS discharge				mRS discharge			
	*F*	*df*	*p*	adj *R*^2^	*F*	*df*	*p*	adj *R*^2^
Overall model	0.593	10,26	0.805	na	0.813	10,23	0.619	na

Medium FIV (15–100 ml)	NIHSS discharge				mRS discharge			
	*t*	*p*	*ß*		*t*	*p*	*ß*	
WholeBrain vessel score	− 5.62	**< 0.001**	− 0.30		− 7.19	**< 0.001**	− 0.37	
Piale collaterals only	− 0.39	0.70	− 0.02		0.1	0.923	0.01	
Atherosclerosis	2.04	**0.04**	0.11		− 0.09	0.926	− 0.01	
Glucose level	2.47	**0.01**	0.12		2.13	**0.034**	0.10	
Statine	− 0.65	0.52	− 0.03		− 1.36	0.174	− 0.06	
Smoking	0.51	0.61	0.02		1.37	0.173	0.07	
Infection	1.07	0.29	0.05		1.76	0.08	0.08	
Age	1.22	0.22	0.06		4.71	**< 0.001**	0.24	
Male sex	0.20	0.84	0.01		0.36	0.72	0.02	
IV thrombolysis	0.65	0.52	0.03		2.43	**0.016**	0.12	
IA thrombolysis (bridging)	0.9	0.367	0.046		1.96	0.051	0.10	
Thrombectomy	2.72	**0.007**	0.131		1.12	0.266	0.052	
Overall model	***F***	***df***	***p***	**adj R**^**2**^	***F***	***df***	***p***	**adj R**^**2**^
	7.224	12,361	**< 0.001**	**0.17**	12.519	12,333	**< 0.001**	**0.29**

**Fig. 2:**
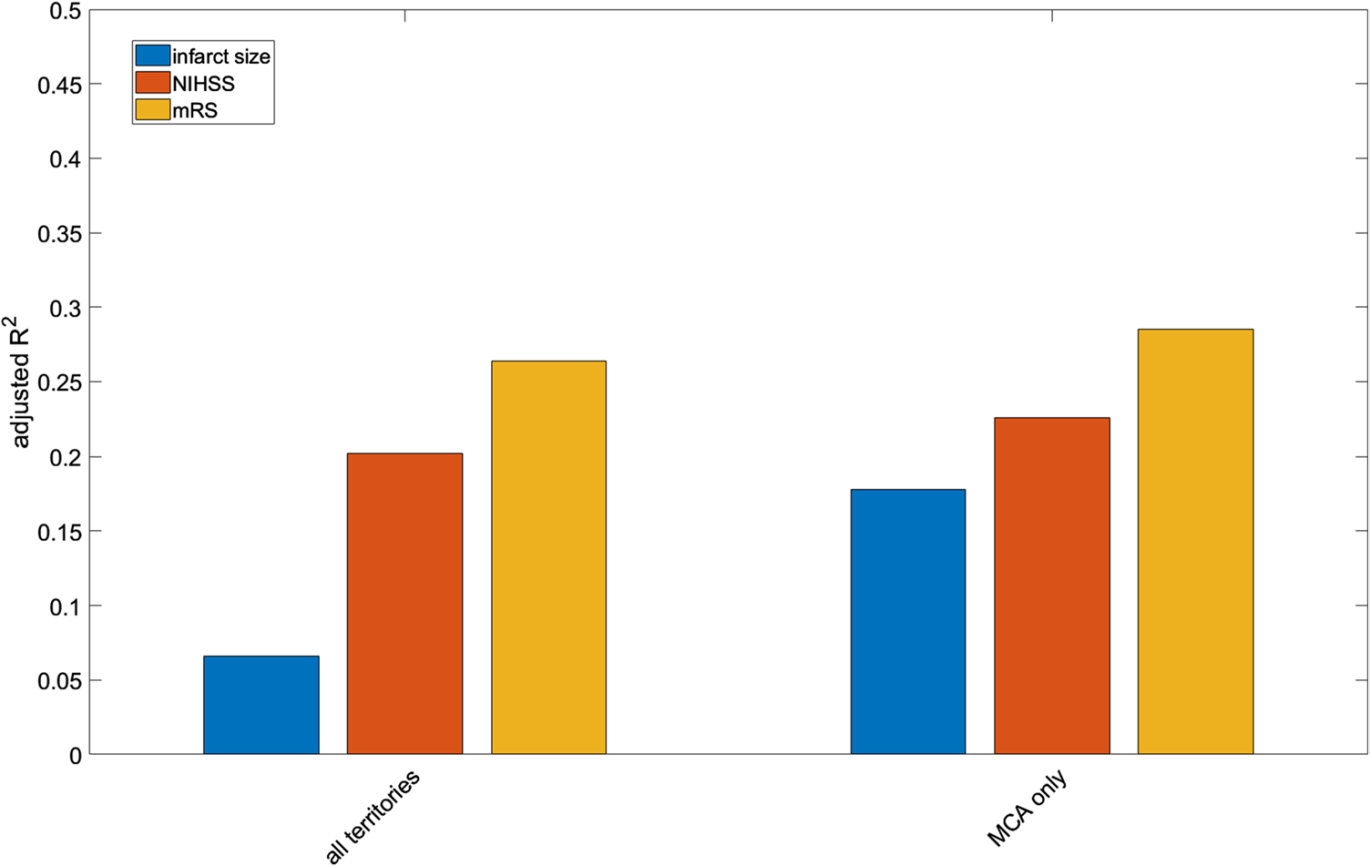
Multivariate analysis of variance (MANOVA) and its effect on the three outcome variables including all data and MCA territory only

In addition, we reanalyzed the data using a MANOVA approach using the same variables as predictors of functional and radiological outcome (see Fig. 2). Using this complementary multivariate analysis approach we could confirm our findings from the original linear regression models. An analysis of medium size and malignant profile strokes was not methodologically possible with this approach. The model was performing best when using mRs at discharge as dedicated outcome variable while it was least predictive of the FIV when considering the whole data set as well as MCA territory strokes only.

## Discussion

We aimed to introduce a simple grading algorithm for collateral vessels in the emergency room setting using the conventional diagnostic measures that are widely available for everyday clinical practice.

On a univariate level we could replicate findings that whole brain collateral vessel status as well as risk factors such as atherosclerosis, elevated glucose levels on admission as well as interventional treatment are related to the functional and radiological outcome of the patients [6–10]. This is of particular interest since we used a novel scoring method to evaluate the collateral vessel status of the whole brain.

However, using a regression model our data show in a large sample of acute stroke patients that the status of the collateral vessels on a whole brain level using CTA is not reliably predictive of the final infarct size, mRs and NIHSS on a multivariate level. This was true for both multivariate analysis approaches used in our study. The predictive capacity of the model was moderately increased when including only strokes in the territory of the MCA. However, with the described model we were able to account for 29% of the variance at most.

Our data shed a new light on several clinical trials that demonstrated the importance of collateral status on clinical outcome using different grading techniques to evaluate patients’ collateral status [11–15]. Most of these studies only included patients with large artery occlusion that were potentially eligible for thrombectomy whereas in our study, median infarct volume and the rate of clinically severe stroke (i.e., NIHSS > 17) was rather small representing an actual natural cohort. There is also great variability in the methods and grading systems used to quantify collateral perfusion that has to be accounted for [4, 16]. In summary, our cohort is far more heterogenous and closer to a real-life scenario because we included all patients admitted with the suspicion of an ischemic stroke/TIA [4, 16–18].

We conclude that a whole-brain vessel score for all patients regardless of the vascular territory involved or the location of the occluded vessel does not appear to help to prospectively identify patients for a more aggressive therapeutic approach. It seems that only the dedicated territorial evaluation concerning the exact location of the ischemic lesion and its surrounding vessels during actual interventional angiography or a combination with perfusion measures would be suitable to better predict the clinical outcome on a multivariate level. It might be possible that grading of the vascular collateral status is more helpful in selected patients with large artery occlusion and/or severe strokes. This question should be addressed in future studies.

It is noteworthy that in our dataset sometimes over 50% of the variance for the respective model could not be explained by any of our variables. This indicates that lot more than the obvious patient inherent factors, such as age and gender or comorbidity, as documented by statin consumption, smoking or infectious complications during the hospital stay and the status of the collateral vasculature seem to contribute to the final infarct size and persisting clinical deficit. Factors that might change the magnitude of cerebral collateral blood flow (genetic or preexisting conditions, actual hemodynamic situation during the CT-angiography) that are not evident at the time of arrival in the emergency department might also play into the relatively low amount of variance explained by our model in general [1, 19].

However, even in the pooled analysis of multiple large multicenter trials of therapeutic interventions on stroke outcome which demonstrated the effect of mechanical thrombectomy on clinical outcome, only 12% of the clinical effect was explained by final infarct volume. Therefore, apart from the unknowns described above this seems to be an inherent limitation of the statistical methods used in our and the aforementioned study to predict outcome based on values of estimation of FIV [20].

Future studies in larger populations should emphasize the role of vascular territory involved and classify the pathophysiological mechanism (large artery occlusion vs. lacunar) in more detail. This has been shown to be effective for large artery atherosclerosis vs. cardioembolic strokes and the effect of adjacent collateral vessels compared with the contralateral non-affected hemisphere. A prediction approach using regional weights should then allow for more precise prediction models than a whole brain analysis since the presence of collateral vasculature is known to differ between spatially distinct regions of the brain [15, 21]. This could help us reach a personalized medicine model in stroke when predicting final infarct size and more importantly clinical outcome by evaluating the status of the collateral vasculature of the brain at event onset.

## Electronic supplementary material

Below is the link to the electronic supplementary material.

Supplementary file1 (DOCX 48 kb)
