# Supplementary material for: Prediction contribution of the cranial collateral circulation to the clinical and radiological outcome of ischemic stroke
Source: J Neurol. 2020 Mar 23;267(7):2013–21. doi: 10.1007/s00415-020-09798-0 (PMC7320948; doi:10.1007/s00415-020-09798-0)
Supplement: Supplementary file 1 — Supplementary file1 (DOCX 48 kb) [file 415_2020_9798_MOESM1_ESM.docx]

**Univariate analysis whole data set**

**Univariate analysis MCA only**
